# The educational needs of people with systemic sclerosis: a cross-sectional study using the Dutch version of the Educational Needs Assessment Tool (D-ENAT)

**DOI:** 10.1007/s00296-015-3352-8

**Published:** 2015-08-31

**Authors:** Anne Schouffoer, Mwidimi E. Ndosi, Thea P. M. Vliet Vlieland, Jorit J. L. Meesters

**Affiliations:** Department of Rheumatology, Leiden University Medical Center, Leiden, The Netherlands; Department of Rheumatology, Haga Hospital, The Hague, The Netherlands; Academic and Clinical Unit for Musculoskeletal Nursing, Leeds Institute of Rheumatic and Musculoskeletal Medicine, University of Leeds, Leeds, UK; School of Healthcare, University of Leeds, Leeds, UK; Department of Orthopaedics, Rehabilitation and Physical Therapy, Leiden University Medical Center, PO Box 9600, 2300 RC Leiden, The Netherlands

**Keywords:** Systemic sclerosis, Patient education, Health knowledge, Outcomes research, Needs assessment, Information

## Abstract

The Dutch Educational Needs Assessment Tool (D-ENAT) systematically assesses educational needs of patients with rheumatic diseases. The present study aims to describe the educational needs of Dutch patients with systemic sclerosis (SSc). The D-ENAT was sent to 155 SSc patients registered at the outpatient clinic of a university hospital. The D-ENAT consists of 39 items in seven domains. “Each domain has different number of items therefore we normalized each domain score: (domain score/maximum) × 100) and expressed in percentage to enable comparisons between domains.” A total D-ENAT score (0–156) is calculated by summing all 39 items. In addition, age, disease duration, gender, educational level, present information need (yes/no) and information need (1–4; wanting to know nothing–everything) were recorded. Univariate regression analysis was used to examine factors associated with the D-ENAT scores. The response rate was 103 out of 155 (66 %). The mean % of educational needs scores (0–100 %; lowest–highest) were 49 % for “D-ENAT total score,” 46 % for “Managing pain,” 41 % for “Movement,” 43 % for “Feelings,” 59 % for “Disease process,” 44 % for “Treatments from health professionals,” 61 % for “Self-help measures” and 51 % for “Support systems.” No associations between the D-ENAT total score and age, disease duration, gender and educational level were found. The D-ENAT demonstrated its ability to identify educational needs of Dutch SSc patients. SSc patients demonstrated substantial educational needs, especially in the domains: “Disease process” and “Self-help measures.” The validity and practical applicability of the D-ENAT to make an inventory of SSc patients’ educational needs require further investigation.

## Introduction

Systemic sclerosis (SSc), or scleroderma, is a complex multiorgan system group of diseases of unknown causative factor associated with increased mortality, complex morbidity, disability and reduced quality of life [1–4]. Despite developments in the medical treatment of SSc, the disease has a major impact on important aspects of patients’ lives [5]. Over the past decades, emphasis has been put on self-management strategies to help patients cope with the consequences of SSc [6]. In order to manage the disease on a day-to-day basis, patients need a formal body of knowledge and skills [7]. Individual and group educational interventions including targeted patient information components have proven to be effective in improving self-management in patients with SSc [8, 9]. Tailored patient education requires a good insight into the individual’s educational needs. Patients with SSc were found to have significant unmet information needs [10], but until recently, no specific instrument was available to systematically assess these educational needs. This hampers the provision of educational strategies that are timely and relevant to the individual patient is difficult and the planning of educational interventions on the group level as well. Recently, the Educational Needs Assessment Tool (ENAT) has been validated as a generic tool to make an inventory of educational needs in rheumatic diseases (including SSc) [11], enabling a systematic assessment and description of the educational needs of people with SSc. This paper aims to describe the educational needs of Dutch patients with SSc by using the Dutch version of the ENAT (D-ENAT).

## Method

### Study design

This was a cross-sectional survey among patients with SSc attending the outpatient clinic of the Department of Rheumatology Leiden University Medical Center in Leiden, the Netherlands (LUMC). The study was judged to be non-medical research by the Medical Ethics Review Committee of the LUMC.

### Patients

In March 2012, 155 adult patients with SSc who visited the outpatient clinic between March 2011 and March 2012 [126 (81 %) women and 29 (19 %) men; mean age 54 years] were identified from the electronic hospital registry. Information leaflets and anonymized questionnaires were sent to the patients via postal mail.

### Assessments

The ENAT has been validated in SSc and other rheumatic diseases [11]. A Dutch version (D-ENAT) has been previously developed and was used in patients with rheumatoid arthritis (RA) [12] and systemic lupus erythematosus (SLE) [13]. The first page of ENAT collects patient-reported information about personal characteristics (age, gender, disease duration and years of education) and two screening questions (1) whether the patient wants to know anything to help cope with a rheumatic disease (yes/no) and (2) an indication of the overall educational need, based on four categories: “I do not want to know anything,” “some things,” “lots of things” and “everything.” The D-ENAT comprises 39 items, grouped into seven domains: Managing pain (6 items), Movement (5), Feelings (4), Disease process (7), Treatments (7), Self-help measures (6) and Support systems (4). Within each domain, patients are asked to rate the question “How important is it for you to know more about….” for all items in that domain. With each item, patients rate their responses using five-point Likert scales with the descriptors: “not at all important” = 0, “fairly important” = 1, “a little important” = 2, “very important” = 3 and “extremely important” = 4. Examples of items in the “Feelings” domain are summarized in Table 1, together with the information of how the ENAT can be obtained. In each domain, the scores of the items are summed to a D-ENAT domain score. The range of domain scores varies from 0–16 to 0–28 depending of the number of items in the domain. The total D-ENAT score is calculated by summing the domain scores (range 0–156), with higher scores representing higher educational needs. Completing the D-ENAT takes approximately 5–10 min [14].

**Table 1:** Example of items in the “Feelings” domain

How important is it for you NOW to know more about the following:	Not at all important (0)	A little important (1)	Fairly important (2)	Very important (3)	Extremely important (4)
Ways to deal with stress	□	□	□	□	□
Ways to deal with moods or depression	□	□	□	□	□
Why I am feeling tired	□	□	□	□	□
Why I am feeling down or depressed	□	□	□	□	□

ENAT can be obtained from the University of Leeds Psychometric Laboratory via the link below: http://www.leeds.ac.uk/medicine/rehabmed/psychometric/Scales1.htm

### Analysis

No missing items were accepted in the calculation of D-ENAT domain and total scores. The missing data were not imputed, and the corresponding domain score was considered “missing completely at random.” Consequently, the estimates of the educational needs were computed using a “complete case analysis” approach [15]. The ordinal D-ENAT data were then transformed into interval level to enable parametric analyses. The conversion tables for the D-ENAT data transformation are published elsewhere [11]. To enable comparisons between domains, domain scores were “normalized” by dividing by the maximum possible score (%) for each domain.

Descriptive statistics were used to analyze the patient characteristics, their D-ENAT domain scores and the total scores. The internal consistency of the D-ENAT domain score and total score was determined by calculating the Cronbach’s alpha. The internal consistency is considered to be good when Cronbach’s alpha is higher than 0.70 [16].

Discriminative validity of the D-ENAT was tested using independent *t* test comparing the total scores for two patient subgroups: those reporting a need for information and those reporting no need for information. In addition, we used one-way ANOVA to compare the D-ENAT total scores across the four different categories of want (“nothing,” “some things,” “lots of things” and “everything”). Post hoc multiple comparisons were made with Bonferroni correction.

Univariate linear regression was used to determine which independent variables were associated with higher educational needs. Regression coefficient *β*-estimates are presented with 95 % CIs. All analyses were performed using SPSS 21.

## Results

### Population characteristics

The questionnaire was returned by 103 patients (66 %) from the cohort of 155 patients, comprising 82 women (81 %) and 19 men (19 %). Their mean (SD) age was 57.5 (13.7) years and disease duration 11.5 (9.9) years, and the duration of education was 11.8 (4.1) years. Eighty-four (82 %) patients completed the D-ENAT without missing items. For the domains, the completion rate was 93–99 (90–96 %).

Regarding self-perceived information need, 27 (27 %) answered “Yes, I want to know anything.” Regarding the magnitude of the information need, nine patients (9 %) indicated that they wanted to know “nothing,” 18 (18 %) “a little,” 26 (26 %) “a lot” and 47 (47 %) “everything.”

### D-ENAT scores

Table 2 shows the educational needs of the patients. The mean (SD) D-ENAT total score was 77.2 (35.4), (49 % of maximal score). The highest educational needs in terms of percentage of the maximum possible domain score were found for “Self-help measures” 14.8 (SD 6.1) (61 % of maximal score) and “Disease process” 16.6 (6.9) (59 %), whereas the average proportional scores for “Treatments from health professionals” 12.5 (8.2) (44 %), “Feelings” 7.0 (4.9) (43 %) and “Movement” 8.2 (6.3) (41 %) were somewhat lower.

**Table 2:** Educational needs among patients with SSc, summarised by D-ENAT domain scores and total scores, with higher scores corresponding to higher educational needs

D-ENAT domains	*n*	Mean (SD)	Mean (% of max.)^a^
1. Self-help measures (0–24)	96	14.8 (6.1)	61
2. Disease process (0–28)	95	16.6 (6.9)	59
3. Support systems (0–16)	96	8.3 (3.7)	51
4. Managing pain (0–24)	96	11.1 (5.2)	46
5. Treatments health professionals (0–28)	93	12.5 (8.2)	44
6. Feelings (0–16)	97	7.0 (4.9)	43
7. Movement (0–20)	99	8.2 (6.3)	41
Total D-ENAT score (0–156)	84	77.2 (35.4)	49

*Mn* mean, *SD* standard deviation

^a^Percentage of maximum possible score (for each domain score or total score)

### Internal consistency and discriminative validity

The internal consistency of the 39 D-ENAT items was good (Cronbach’s Alpha = 0.97).

The discriminative validity of the D-ENAT was demonstrated by comparing total scores of two subgroups of patients: those with a present information need and those without. The difference of mean total scores for the two subgroups were statistically significant, respectively, 102.4 (S.D. 31.8) and 68.2 (S.D. 32.3) (*t* = 4.27, *df* = 82, *p* < 0.001).

The discriminative validity of the D-ENAT was further reflected by the total score which increased with the categories of the patient’s general information need. The mean D-ENAT total scores (SD) were 54.9 (43.7), 51.3 (23.6), 76.4 (26.6) and 93.7 (35.4) in the first, second, third and fourth categories of the general information need question, respectively. The differences seen in the D-ENAT total scores between the first and the fourth (*p* = 0.025) and the second and the fourth (*p* < 0.001) categories were statistically significant (with Bonferroni correction).

### Independent variables associated with higher educational needs based on D-ENAT total score

There were two significant associations among determinants of educational needs: age with duration of education (Spearman’s-rho (rs) = 0.31, *p* < 0.01) and age with disease duration (*r*s = −0.35, *p* < 0.01). Other correlations were substantially lower and not significant.

Table 3 shows the results of the linear regression analysis for the D-ENAT total score and D-ENAT domain scores as dependent variables and all independent variables (i.e., age, gender, disease duration and duration of education). Age, gender, disease duration and duration of education were not associated with the D-ENAT total scores. However, longer duration of education was associated with lower educational needs within two domains: “Movement” (*β*-est = −0.4; 95 % CI −0.7, −0.08) and “Treatment from other health professionals” (*β*-est = −0.5; 95 % CI −0.9, −0.08).

**Table 3:** Univariate linear regression of D-ENAT total score (dependent variable) and all independent variables entered separately in the model

D-ENAT domain (range)	Being female	Age (years)	Disease duration (years)	Education (years)
Total (0–156)				
*β*	5.8	−0.09	−0.3	−1.5
95 % CI	−14.4, 26.1	−0.7, 0.5	−1.1, 0.4	−3.4, 0.4
Pain (0–24)				
*β*	0.3	−0.01	−0.03	−0.24
95 % CI	−2.8, 2.8	−0.09, 0.08	−0.01, 0.08	−0.5, 0.02
Movement (0–20)				
*β*	0.8	0.2	−0.03	−**0.4**
95 % CI	−2.5, 4.1	−0.07, 0.11	−0.2, 0.1	−**0.7**, −**0.08**
Feelings (0–16)				
*β*	0.7	0.02	−0.007	−0.2
95 % CI	1.9, 3.3	−0.06, 0.09	−0.11, 0.10	−0.4, 0.09
Disease process (0–28)				
*β*	−1.0	0.003	−0.09	−0.3
95 % CI	−4.6, 2.6	−0.1, 0.1	−0.2, 0.05	−0.7, 0.04
Treatments (0–28)				
*β*	0.4	0.07	−0.03	−**0.5**
95 % CI	−3.9, 4.7	−0.06, 0.2	−0.2, 0.1	−**0.9**, −**0.08**
Self-help (0–24)				
*β*	0.5	−0.04	−0.05	−0.06
95 % CI	−2.7, 3.8	−0.13, 0.06	−0.2, 0.1	−0.4, 0.2
Support systems (0–16)				
*β*	−1.0	−0.01	−0.03	−0.02
95 % CI	−3.0, 0.9	−0.06, 0.05	−0.1, 0.05	−0.2, 0.2

Bold numbers indicate significant associations, *p* ≤ 0.05

## Discussion and conclusions

This cross-sectional study showed that patients with SSc have considerable educational needs. The mean total score on D-ENAT was 77 (49 % of maximum scoring), and relatively high scores were found for the domains “Disease process” and “Self-help measures.” There was no association between the overall educational needs and patient characteristics except for two domains: “Movement” and “Treatments from other health professionals,” where high education need scores were associated with fewer years of education.

The educational needs of patients with SSc from our hospital were lower than patients with systemic lupus erythematosus (SLE) with a normalized D-ENAT score of, respectively, 49 and 56 %. The lower educational need was most prominent within the domains “Movement,” (−9 %) “Feelings” (−15 %) and “Treatments from other health professionals” (−12 %) [13]. Comparisons between SSc and RA patients from the Netherlands cannot be made due to recent differences in D-ENAT scoring [11].

In contrast with other questionnaires, the ENAT is specifically designed to identify both the extent of information need as well as the domains in which information is desired. Two other questionnaires which incorporate some aspects of educational needs are Systemic Sclerosis Needs Questionnaire (SSNQ) [17] and a survey on the need for a hospital-based Interactive health Communication Application [18]. In the UK, Rubenzik et al. [17], using the SSNQ, found the highest prevalence of unmet needs in the psychological/spiritual/existential domain. In contrast, a survey in the Netherlands [10] using the same questionnaire found most unmet needs on disease-related medical topics such as medical tests and treatment. Van der Vaart et al. [18] using a survey on the need for a hospital-based Interactive health Communication Application found that the need for online information was high on various subjects: “scientific research,” “overview of the disease,” “treatment options” and “physical consequences of the disease.” These results are partly in line with the results of the present study demonstrating high scores in “Disease process” and “Self-help measures” domains. Based on these studies, we can conclude that information needs among patients with SSc are high and areas in which information is needed are predominantly concerning medical information.

The importance of patient education as identified in our study was confirmed by the focus group study in patients with SSc by Mendelson et al. [19]. They reported that access to high-quality, timely information provides patients with the tools to develop and implement a strategy of self-advocacy in disease management. This is in line with the highest educational needs seen in the domain “Self-help measures” in our D-ENAT study. In our study, we found no correlation between information need and patient characteristics. In contrast, Schouffoer et al. [10] found that higher information need was associated with having diffuse SSc, having a partner and a lower physical component summary scale SF-36. Rubenzik et al. [17] found that patients with lower education levels were more likely to report unmet needs in health information. Differences may be explained by the relatively small study samples. Nevertheless, it is suggested that healthcare providers should be aware of subsets of patients who are more in need of information than others (i.e., patients with more physical disability or a lower education level)).

This study had three main limitations. First, the ethical approval for our study did not allow for a comparison between responders and non-responders since the questionnaires were anonymized. The results of this study, therefore, need to be interpreted with care, as selection bias cannot be completely excluded. However, the age and gender of the 155 eligible (responders and non-responders) patients (81 % female and mean age 54 years) were similar to those of the 122 responders (81 % females, mean age 58 years). Given the fact that we also achieved a 66 % response rate, despite the inability to send reminders, the selection bias is likely to be limited. Moreover, compared to other studies with an observational design, the disease duration of 11.5 years in our sample was not abnormal, however, relatively high [20]. Therefore, it could be hypothesized that we underestimated the educational needs among patients with SSc, since shorter disease duration is associated with higher educational needs. Second, the sample was selected from a SSc-specialized hospital; therefore, the generalizability of the results may be limited. For example, a considerable geographical variation in patient satisfaction with healthcare experience [21] was demonstrated in the UK. Third, although educational needs are clearly present, 27 % of the patients expressed either no or little educational needs. Although this could be caused by a true absence of explicit educational needs regarding SSc and its treatment, it could also be caused by other factors that cannot be explained by this cross-sectional survey.

In conclusion, the results of this study have shown that the educational needs among Dutch-speaking SSc patients are substantial and can be reliably assessed by the D-ENAT. The findings underline the importance for patient education in SSc as an integrated part of standard rheumatologic care. Given the emphasis on patient-centered health care and tailor-made educational programs [22, 23], the identification of individual educational needs becomes imperative. To our knowledge, the D-ENAT is the only objective tool available to fully explore the individual educational needs among Dutch-speaking patients with SSc. Further research into the usefulness of the D-ENAT in daily clinical practice and its application in other rheumatic diseases is needed.
